# Learning from multiple readings for axial spondyloarthritis classification of the sacroiliac joints

**DOI:** 10.1038/s41598-026-39417-3

**Published:** 2026-02-19

**Authors:** Amir Jamaludin, Rhydian Windsor, Sarim Ather, Gregory Ligozio, Aimee Readie, Pedro M. Machado, Timor Kadir

**Affiliations:** 1 Visual Geometry Group, Department of Engineering Science, University of Oxford, Oxford, UK; 2 Oxford University Hospitals, Oxford, UK; 3 Novartis Pharmaceuticals, East Hanover, USA; 4 Department of Neuromuscular Diseases, UCL Queen Square Institute of Neurology, University College London, London, UK; 5 National Institute for Health Research (NIHR) University College London Hospitals Biomedical Research Centre, London, UK; 6 Department of Rheumatology, Northwick Park Hospital, London North West University Healthcare NHS Trust, London, UK; 7 Plexalis, Oxford, UK

**Keywords:** MRI, Spine, Sacroiliac joint, Ankylosing spondylitis, Spondyloarthritis, Image processing, Machine learning, Predictive medicine

## Abstract

Magnetic resonance imaging (MRI) is a cornerstone in the evaluation and monitoring of axial spondyloarthritis (axSpA), a chronic inflammatory condition primarily affecting the sacroiliac joints (SIJs), spine, entheses, and peripheral joints. Accurate quantification of axSpA-related changes on MRI is critical for effective research and patient management; however, current lesion detection and grading approaches suffer from substantial intra- and inter-reader variability, limiting their consistency and reliability. To address these challenges, we propose a fully automated machine learning system for SIJ delineation and lesion classification on coronal MRI. The end-to-end pipeline automatically extracts SIJ contours using a vector-field—based open-contour model and classifies the presence or absence of five lesion types (bone marrow oedema, ankylosis, sclerosis, erosions, and fatty lesions) using both T1-weighted and STIR sequences. A multi-reader learning framework is employed to explicitly model inter- and intra-reader variability by leveraging multiple readings and consensus labels. Model performance was evaluated using patient-wise cross-validation on data from the MEASURE-1 clinical trial and further validated on other clinical datasets (PREVENT, SURPASS). Lesion classification performance was assessed using area under the receiver operating characteristic curve (AUC), balanced accuracy, sensitivity, and specificity, while contouring accuracy was quantified using root-mean-square error, where we found that 95% of the whole test set had errors below 2.76mm. The proposed approach achieved AUCs ranging from 0.85 to 0.99 across the five lesion types, with the highest performance observed when using consensus-based labels, and results were comparable to expert inter-reader agreement. These findings demonstrate that fully automated SIJ delineation and lesion scoring can achieve expert-level performance and have the potential to reduce reader burden and variability in large-scale axSpA MRI studies.

## Introduction

Magnetic resonance imaging (MRI) is a commonly used method for supporting the diagnosis, as well as assessing and monitoring the progression, of axial spondyloarthritis (axSpA), a chronic inflammatory rheumatic disease that affects the sacroiliac joints (SIJs), the spine, entheses, and peripheral joints^1–4^. Detection and quantification of axSpA-related changes in MRI have become valuable tools in patient management. However, like many other imaging detection and grading schemes for various diseases, these evaluations remain prone to intra- and inter-reader variability, making it challenging to train a single model to detect and grade axSpA-related MRI changes^5^.

In this work, we specifically focus on the sacroiliac joints and the automatic detection of these changes. This paper proposes a model training strategy that learns from multiple labels provided by multiple readers, where a single reader possesses multiple potential labels. As mentioned, this is applied to the task of grading axSpA-related MRI lesions^6^, specifically: (i) bone marrow oedema, (ii) fatty lesions, (iii) erosions, (iv) sclerosis, and (v) ankylosis.

### Related work

There have been multiple works on detecting or segmenting parts of the spine in spinal medical imaging across several imaging modalities, e.g. intervertebral discs ^7^ and vertebral bodies in MRI ^8^ and CT scans ^9^ as well as the whole spine in DXA scans ^10,11^. However, there has been relatively little research on detecting the SIJ and related downstream tasks, for example, inflammation prediction or quantifying structural changes. The closest work to date on SIJ delineation is^12^. However, this method focuses on the classification of sacroiliitis and requires manual annotation to locate the SIJ region. Another closely related work is^13^, where the authors propose a method to detect changes in the SIJ. However, this is done without explicitly focusing on the SIJ region, instead taking the whole slice of an SIJ MRI as input. We propose that by delineating the SIJ, models can focus on the exact region of the disease without additional noise from surrounding anatomical structures.

Our contouring method has analogies to several works on shape representation using deep learning via implicit functions (e.g. ^14–16^). In this case, rather than representing shapes as a binary mask over a regular grid of voxels, a model learns $f: \mathbb {R}^3 \rightarrow \mathbb {R}$, such that *f*(*x*, *y*, *z*) estimates the closest distance from point (*x*, *y*, *z*) to the object of interest’s surface (*signed distance* functions), or whether (*x*, *y*, *z*) is occupied by the shape (*occupancy* functions). These methods allow for sub-pixel/voxel precision representations of surfaces. Though we validated our approach on SIJ MRIs, it is worth noting that open contours are widely used in other medical imaging tasks e.g. torso contour segmentation for better ECG interpretation^17^, and reconstructing 3D meshes of the heart from 2D cardiac MRIs^18^.

In terms of lesion detection, there are several works on machine learning systems detecting inflammation on the sacroiliac joints (SIJs) which can be broken down into two separate categories, namely: (i) semi-automatic pipeline needing humans in the loop by Kucybała et. al.^19^, Zarco et. al.^20^, and Garrido-González et. al.^21^, and (ii) fully-automatic pipeline which is similar to our approach but looking specifically at the SIJs by Ożga et. al.^22^, Rzecki et. al.^23^, Bressem et. al.^24^, Lee et. al.^25^, and Nicolaes et. al.^26^. It is worth noting that none of these works delineate the SIJs before lesion detection, and none assessed the SIJs regarding the presence of the wide range of acute and chronic lesions predicted in our pipeline.

### Method overview

There are two main stages to the pipeline: (i) the first stage is to detect or delineate the sacroiliac joints (both left and right joints) in a given MRI volume^27^, and (ii) the second stage is to detect all the MRI lesions associated with axSpA. We discuss these parts separately but at inference time, the delineation and classification stages are combined to process a given SIJ MRI.

## Delineating the SIJs

Contouring objects is a very important step in various medical image analysis tasks. Currently, one common approach is to predict a segmentation map of the object and then extract the map’s edges. However, this approach has limitations. Firstly, the output segmentations are not necessarily a single interconnected volume and thus additional post-processing is required before finding edges, which can introduce errors (e.g. by removing additional volumes). Secondly, this method does not allow for detecting open contours. An alternative approach is to treat pixels along the open contour as segmentation targets. However, this approach often leads to small, challenging segmentation targets. Furthermore, these approaches do not guarantee a unique solution or easily allow for sub-pixel precision contours in both the open and closed settings.

Therefore, we propose to delineate contours, avoiding these limitations. This is done by ‘walking’ along a learnt vector field. Along the contour, the field should point parallel to the contour, whereas outside the contour the field should point to the nearest contour point. To demonstrate the effectiveness of this method, we apply it to a novel task; delineating the sacroiliac joint (SIJ) boundary in clinical MRI scans.

The SIJ is the joint between the sacrum of the spine and the ilia of the pelvis. There are two SIJs per person, one on the left and one on the right. MR imaging is typically done to look at the inflammation of the SIJ, or sacroiliitis, which is one of the causes of low back pain and an important element to support the diagnosis of axial spondyloarthritis (axSpA). axSpA SIJ MRI evaluation methods often refer to specific regions surrounding the SIJ^28^, which makes SIJ detection a must. Since the SIJ is defined as the space between two bones, we follow the approach suggested by^29^ and delineate each SIJ as an individual open contour, which is beneficial for the further downstream task of grading the SIJ. Our approach to delineate the SIJ was presented in ISBI 2023^27.^

**Fig. 1:**
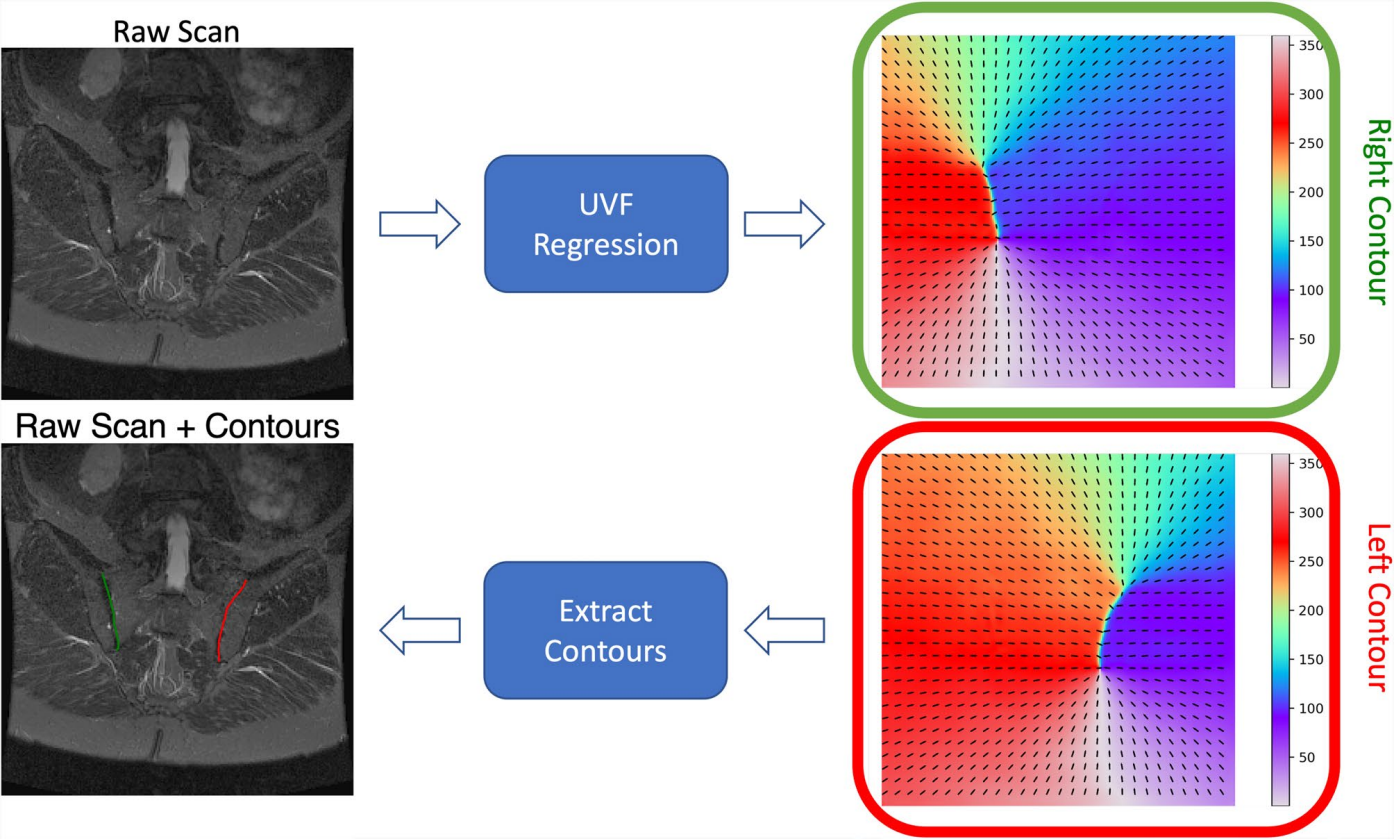
Overview of the contouring pipeline on an example SIJ MRI. The model outputs two vector fields, one each for the left (red) and right (green) SIJs. Each vector field is shown as a gradient map of the angle (in degrees) of the vector at that point. These vector fields are then used to extract contours for both the SIJs, shown in the bottom left panel.

### Approach overview

Our method takes as input 2D images and outputs an array of vertices delineating the contour of interest. This is done in two main steps: (i) a model to predict *a unit vector field* (UVF) for the image. At location ${\bf x}$, the UVF indicates the direction towards the nearest point on the contour of interest; (ii) a method to extract open contours from this learned vector field. Our overall approach for the task of SIJ delineation can be seen in Fig. 1.

**Fig. 2:**
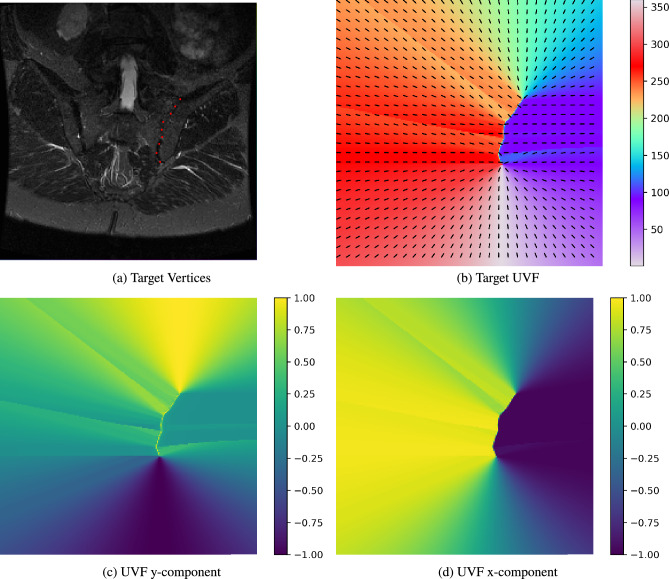
The unit vector field (UVF): (**a**) a slice of an SIJ MRI with annotated landmarks in red delineating the left SIJ (with respect to the patient orientation), (**b**) the resulting target UVF, overlaid on top of a gradient map of the field’s direction in degrees, (**c**) y direction of the UVF, and (**d**) x direction of the UVF. (**c, d**) Values range from $-1$ (blue) to +1 (yellow).

### Unit vector fields

The idea of contours and vector fields in combination is not a new one; for example, several early works in computer vision combined Snakes^30^ with gradient vector flow^31^, i.e. a vector field pointing towards object edges in a given image. However, instead of defining the vector field using object edges, we instead learn the unit vector field, $\boldsymbol{\hat{v}}_{i,j}$, where at each location in the vector field, (*i*, *j*), the field ‘points’ to the nearest vertex, i.e. annotated ground truth landmark, on the contour of the object; here *i* and *j* are the pixel coordinates. The unit vector field is made of two separate x and y components corresponding to the directions of the vectors in the field. To preserve the directionality of the contour, we impose a rule where vectors lying on top of the contour should ‘point’ to where the next vertex is expected. The vector fields are normalized such that the magnitude of a given vector, $\boldsymbol{{v}}_{ij} = (x_i, y_j)$, is 1 i.e. $|\boldsymbol{{v}}_{ij}| = 1$. An example unit vector field can be seen in Fig. 2.

**Fig. 3:**
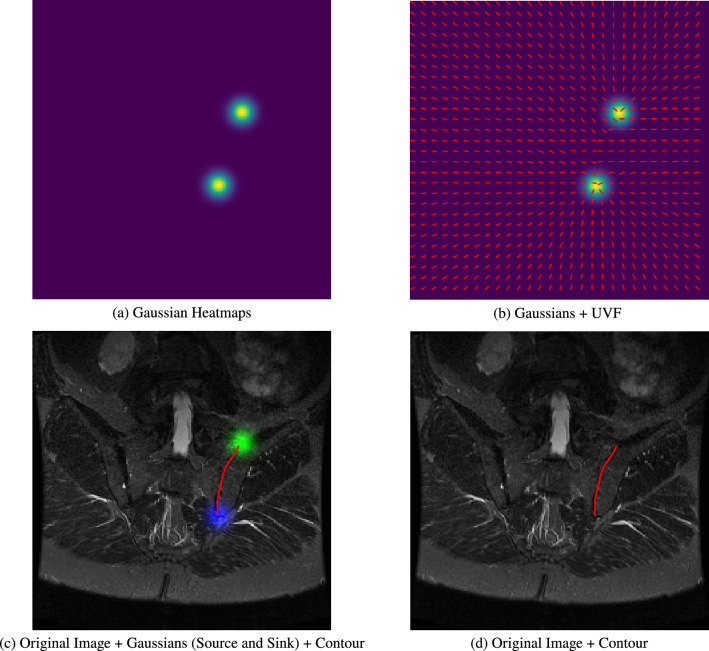
Following on from the example shown in Fig. 2, alongside the UVF, we regress two 2D Gaussian heatmaps. (**a**) 2 Gaussians representing the start and end points of the contour, (**b**) the UVF overlaid on top of the Gaussians, (**c**) the contour which starts from the Gaussian now marked in green and ends on the Gaussian marked in blue, (**d**) the final contour for the left SIJ marked in red.

### Extracting contours from unit vector fields

**Fig. 4:**
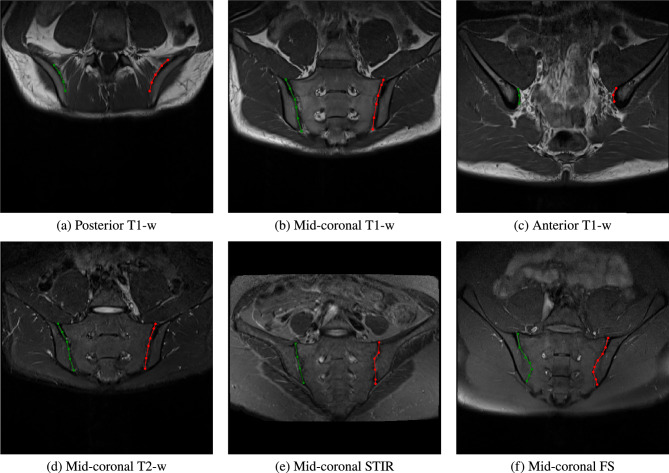
Example scans in the dataset with their marked-up annotated landmarks. (**a–c**) Slices from the same T1-weighted scan at differing slice positions (anterior, mid-coronal, posterior) while (**d–f**) are mid-coronal examples of different sequences in the dataset.

The unit vector field alone does not obviously indicate where a contour starts and ends. We solve this by also predicting the start and end points with the same network that generates the unit vector field; this is done simultaneously as a separate output. We take inspiration from previous works^8,32,33^ and regress two distinct Gaussian heatmaps for the start and end points respectively. Each Gaussian has a maximum value of 1 and a variance proportional to the area of the task-relevant object. The Gaussian heatmaps are essentially a pair of ‘source’ and ‘sink’ nodes signifying the start and end points of a given contour. In our case, we use the sacrum, i.e. the area which lies in between two SIJs. In the case where the contour is without a defined area of interest, we scale the Gaussian heatmap proportional to the length of the overall contour. The beginning of the contour is defined from the Gaussian heatmap designated as the start point. We then iteratively ‘walk’ following the direction in the UVF, $\mathbf {\hat{v}}_{i,j}$, and the contour ends when approaching the second Gaussian heatmap, i.e. the end point. Each step is 1 unit in magnitude, although this could be adjusted to generate contours of varying fidelity. Figure 3 gives an example of how a contour is defined with the Gaussian heatmaps and the UVF. Since the UVF can be visualized, errors can be more easily interpreted. Though not shown in this work, a closed contour solution would not require heatmaps and could be found by simply searching for a loop in the UVF. In summary, to delineate the contour of the SIJ, we merely draw from ‘source’ to ‘sink’ following the direction of the vector field. Note that, in our use case the SIJs are delineated in every slice for a given 3D volume (Fig. 4).

### Dataset

The Oxford Sacroiliac Joint (**OSIJ**) dataset is a collection of SIJ MRIs from 339 patients that were scanned in the Oxford University Hospitals NHS trust.

For experiments conducted in this work, the dataset is split into training (80%), validation (10%), and testing (10%) sets on a per-subject basis (271:34:34). Each subject possesses an average of two sequences (typically T1, T2, STIR, and FS) resulting in a total of 793 scans. Each scan consists of approximately 20 2D slices, resulting in a total of 16,978 images.

For the annotations of the contour of the SIJs, an expert was tasked with marking the landmarks (vertices) that best define both left and right SIJs through every slice in a given scan. The number of landmarks varies depending on the view of the SIJ; typically, mid-coronal SIJs cover a bigger image area demanding a larger number of landmarks and vice versa. The number of landmarks per slice ranges from 2 to 21.

**Fig. 5:**
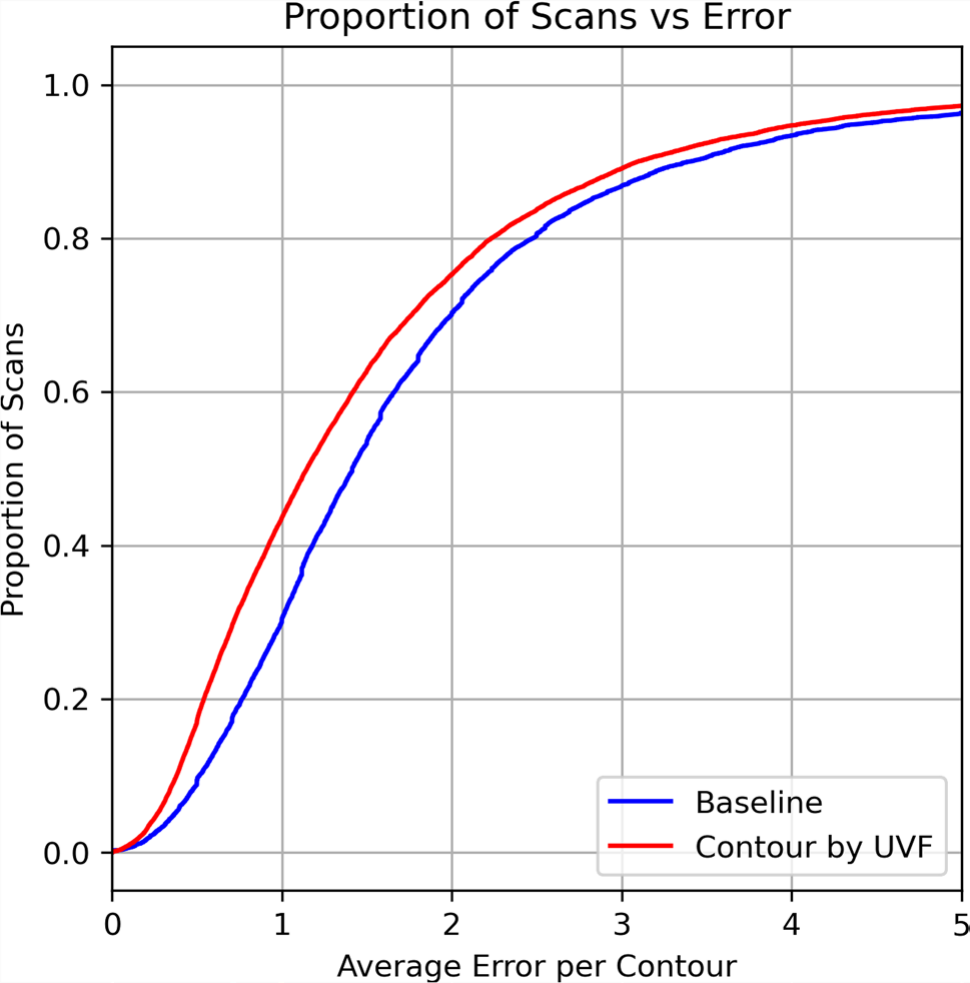
Cumulative test set error distribution (measured in pixels). Baseline is in blue and contouring via UVF is in red.

### Training details

The experiments in this work were conducted using a simple U-Net architecture ^34^. For each contour, the network predicts 2 Gaussian heatmaps and 2 components (x and y direction) of the unit vector field; separate contours were predicted for each of the two SIJs (left and right). The SIJs are not guaranteed to be inside the field-of-view of the scans and as such these cases were kept in the training set to suppress false positives. The scans were typically squares in shape; thus, they were bi-cubically re-sampled to $224 \times 224$ pixels. Slices that were not square were padded with zeros prior to re-sampling so as to not change the aspect ratio. In our pipeline, the delineating network predicts both the left and right SIJs simultaneously, resulting in two separate ‘source’ Gaussians, ‘sink’ Gaussians, and unit vector fields.

The network is trained using an Adam optimiser^35^ with $\beta _1 = 0.9$, $\beta _2 = 0.999$ and a learning rate of $10^{-3}$ until convergence. Several augmentations were applied during training, namely: (a) translation $\pm 20 \%$, (b) scale $\pm 20\%$, (c) rotation $\pm 15^\circ$, (d) left/right flips, (e) additive Gaussian noise, and (f) Gaussian blur. A combination of L2 loss, for the UVF, and weighted L2 loss (see ^8^), for the Gaussian heatmaps, is used to train the network.

**Fig. 6:**
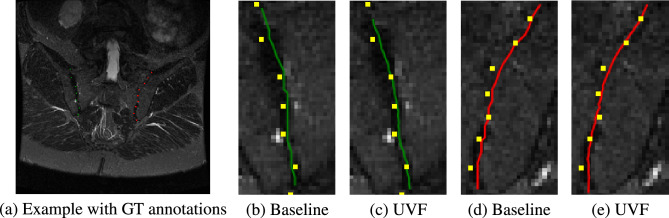
Quantitative result of the baseline against the proposed method on a test set example. **(a)** Example shown with GT landmarks, green for the right sij and red for the left. For **(b, c, d, e)** green contours highlight the right SIJ and red contours highlight the left; GT in yellow. (**b, d**) show results from the baseline model while (**c, e**) are contours using UVF. Baseline predictions are sparse, with 21 landmarks for each contour, resulting in more aliasing.

**Fig. 7:**
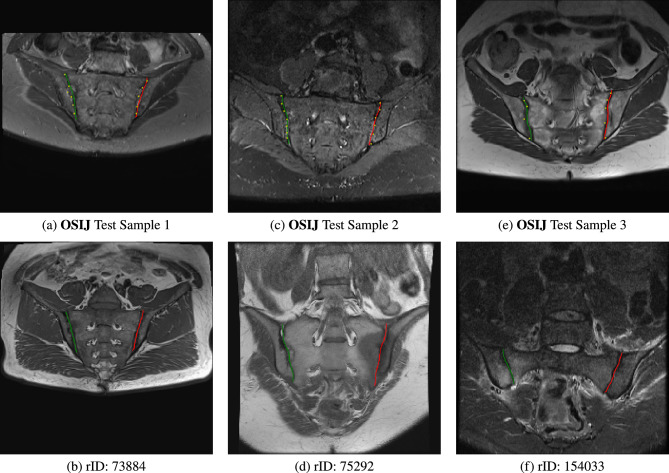
Example contours via UVF. (**a, c, e**) are from the **OSIJ** test with ground truth annotations in yellow (**b, d, e**) are real-world unseen samples taken from Radiopaedia (73884, 75292, 154033).

### Performance evaluation & results

For comparison, we evaluate against a baseline network trained to predict 21 Gaussian heatmaps for each SIJ, 21 being the maximum number of landmarks in the dataset. We find this to be the simplest naïve solution to predict landmarks using a similar U-Net architecture as our proposed UVF approach. Samples with a lower number of annotated points were up-sampled via linear interpolation. At test time, each prediction is compared against the ground truth landmarks of the contour and the root-mean-square (RMS) error is calculated from the closest points between the prediction and ground truth.

Results for both networks are shown in Fig. 5 and Table 1. Contouring by UVF overall works better than the baseline ranging from 0.14 to 0.35 difference in RMS pixel error up to $95\%$ of the data in the test set. Although not a large amount quantitatively, Fig. 6 highlights that there is in fact lower aliasing when looking at the contours using UVF compared to just predicting landmarks via heatmaps. In general, $95\%$ of the test set has a lower than 4.10 pixel error (2.76 mm) which for our purposes is adequate for further downstream tasks, i.e. defining an ROI for SIJ grading. Figure 7 shows results on several examples both from the **OSIJ** test set and to images extracted from Radiopaedia.

**Table 1:** Table of RMS per proportion of data in the test set in terms of pixels (mm in brackets).

Data proportion		0.1	0.3	0.5	0.7	0.9	0.95
Baseline error		0.52 (0.29)	1.00 (0.57)	1.41 (0.86)	2.00 (1.27)	3.40 (2.24)	4.45 (2.98)
UVF error		0.38 (0.21)	0.72 (0.43)	1.15 (0.69)	1.76 (1.10)	3.10 (2.05)	4.10 (2.76)

## Classifying the SIJs

**Fig. 8:**
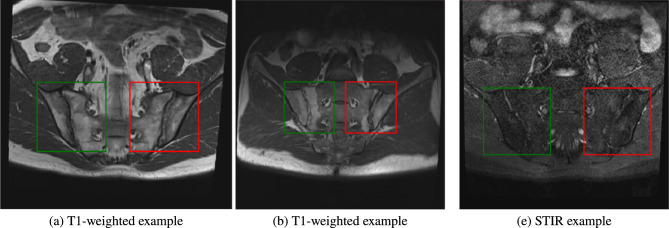
Example ROIs in different scans in the dataset with their marked-up annotated landmarks. (**a, b**) T1-weighted examples while (**c**) is a STIR example. Depending on the FOV of the scan, the SIJs can vary in size which is why ROI detection is crucial in our case. For example, the SIJ in (**b**) is relatively smaller when compared to the SIJs in (**a, c**). The right SIJs are shown in green bounding boxes while the left SIJs are shown in red bounding boxes. Although we show a single slice here, the ROIs cover the 3D volume of a given scan sequence (T1-weighted or STIR) but remain static in spatial coordinates.

Once the SIJs have been delineated, we extract the primary ROIs for the classification tasks. We do this by first determining the upper and lower bounds of the left and right SIJs over every single slice in the volume and rotate the volume in the coronal plane such that the lines between the upper left and right, and lower left and right SIJs are close to horizontal. Then the first ROIs detected are the two separate bounding boxes, each for the left and right SIJ. These boxes are minimum-bounding boxes that cover the SIJ delineation without any margin over the whole slices given a scan. Each ROI is made to be consistent slice-wise for a given volume. An example of the final ROI on the volumes in **MEASURE 1** can be seen in Fig. 8

### The SIJ lesions

We train a classification model to classify five main lesions of the SIJs, using the SIJ ROI as input. The five lesions are: (i) bone marrow oedema, (ii) fatty lesions, (iii) erosions, (iv) sclerosis, and (v) ankylosis. Lesion classification was performed at the quadrant level of each SIJ for bone marrow oedema, fatty lesions, and erosions, and at the SIJ level (left and right) for sclerosis and ankylosis. The quadrants are: (i) upper ilium, (ii) lower ilium, (iii) upper sacrum, and (iv) lower sacrum. As such, for a given ROI, the left SIJ, for example, we produce 14 lesion predictions per SIJ (3 lesions times 4 quadrants plus 2 lesions) resulting in 28 predictions for a given pair (left and right) of SIJs. The lesion predictions are all binary. Each input to the classification model is a pair of ROI volumes from a given SIJ detected from the T1-weighted and STIR sequences for a given patient. Each lesion type was labelled by two expert readers and three independent read sessions except for in **SURPASS,** where there were three expert readers and one read session. The lesions were graded using the Berlin SIJ scoring system assessment criteria^5,36^.

### The classification pipeline

This classification model is a convolutional neural network which is a ResNet34^37^ model pre-trained for other spinal diseases^38^. The model takes as input two sequences, T1-weighted and STIR, for each SIJ. The model uses the same encoder for each input sequence and the resulting pair of embeddings were concatenated before subsequent lesion-type-specific linear layers. In total, there are 14 separate linear layers from a given pair of embeddings.

Since we possess multiple labels for every lesion type, learning the best prediction for a given input is not straightforward. The simplest approach would be to use a “consensus” label using all the available labels; we have 2 readers with 3 labels each (from independent read sessions and the readers are blinded from their previous labels as appropriate for a clinical trial). This results in 3 good plausible “consensus” labels: (i) inter-consensus labels where a label is only used when between BOTH readers at ALL read sessions, (ii) intra-reader consensus labels for reader 1, i.e. labels are only used where reader 1 agrees with themselves over 3 read sessions, and (iii) an intra-reader consensus for reader 2.

**Fig. 9:**
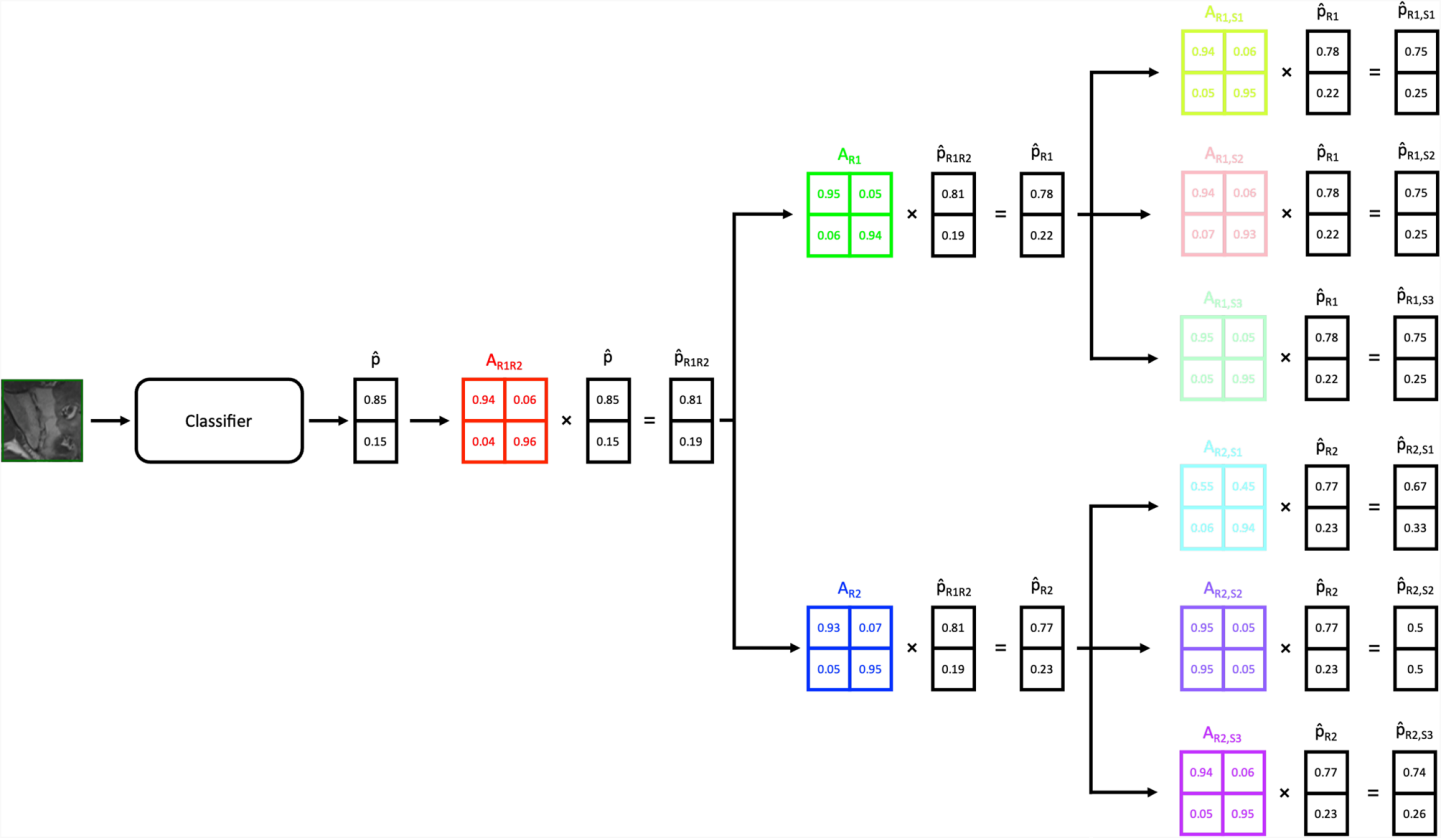
Example showing an SIJ as input. The immediate output $\boldsymbol{\hat{p}}$ is then transformed into the inter-consensus prediction via matrix multiplication with the inter-consensus annotator matrix $A_{R1R2}$ (red). Subsequently, the prediction is then further multiplied with specific intra-reader annotator matrices $A_{R1}$ (green) and $A_{R2}$ (blue) resulting in two separate intra-reader specific predictions. The intra-reader predictions are then further split into session-specific readings S1, S2, and S3 for all 3 read sessions. The example shown corresponds to one prediction but in practice, we perform 14 separate predictions (3 lesion types (Oedema, Erosions, Fatty Lesions) with 4 quadrants plus 2 for Ankylosis and Sclerosis). Also, note that the input slice shown is a single 2D slice from a sequence but in actuality, we process all T1-weighted and STIR slices in combination for a single SIJ. At inference time, $\boldsymbol{\hat{p}_{R1R2}}$ is used as our best prediction when comparing against inter-reader labels.

We follow the method suggested by Tanno et al. ^39^ with a few modifications that are relevant for training a model with multiple readers where a single reader can provide multiple labels (at different annotation sessions). Tanno et al. ^39^ described a simple method to train a model from multiple annotations essentially by training annotator matrices, $\boldsymbol{A}_r$, that shifts a prediction, $\boldsymbol{\hat{p}(x)}$, where r represents a specific annotator and *x* is the input, such that the final prediction can be annotator specific $\boldsymbol{\hat{p}_r(x)} = \boldsymbol{A}_r \boldsymbol{\hat{p}(x)}$. Three tweaks that we implemented in our approach are: (i) instead of learning confusion matrices $\boldsymbol{A}_r$ where r represents the readers; we instead learn two vectors, $\boldsymbol{a}_r$ and $\boldsymbol{b}_r$, where the vectors can be expressed as $\boldsymbol{a}_r \otimes \boldsymbol{b}_r = \boldsymbol{A}_r$ akin to a LoRA^40^ approach of rank 1, (ii) instead of using $\boldsymbol{A}_r$ directly with the prediction we instead use $\boldsymbol{\hat{p}_r(x)} = \boldsymbol{B}_r\boldsymbol{\hat{p}(x)}$ where $\boldsymbol{B}_r = {\bf I}+\boldsymbol{A}_r$ and ${\bf I}$ is the identity function, and (iii) instead of predictions being essentially independent of one another, our predictions are based on the hierarchy of “consensus” of the labels and as such we branch our predictions as follows:


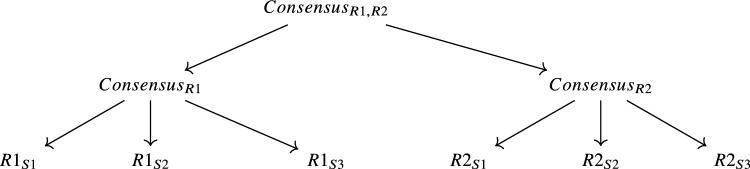


where $Consensus_{R1,R2}$ is the inter-reader consensus of the two readers at ALL 3 read sessions, $Consensus_{R1}$ and $Consensus_{R2}$ are the intra-reader consensus of the two readers at ALL 3 read sessions, and $R1_{S1}$, $R1_{S2}$, $R1_{S3}$, $R2_{S1}$, $R2_{S2}$ and $R1_{S3}$ are labels of a specific reader at a particular read session. When assessing the predictions against the ground truth we use the inter-reader labels (the consensus label of all the readers at ALL read sessions), i.e. $Consensus_{R1,R2}$, to measure our performance but in training all the labels are used according to Fig. 9. We follow the loss formulation of Tanno et al. ^39^, which combines the cross-entropy loss with a regularisation term given by the sum of the traces of the annotator matrices:

$$\mathcal {L} = \mathcal {L}_{\textrm{CE}} + \sum _{i \in \mathcal {I}} \operatorname {tr}({\bf A}_i),$$

where $\mathcal {I} = \{\text {R1S1}, \text {R1S2}, \text {R1S3}, \text {R2S1}, \text {R2S2}, \text {R2S3}, \text {R1}, \text {R2}, \text {R1R2}\}$, and ${\bf A}_i$ denotes the annotator matrix corresponding to reader/session *i* or consensus.

### Datasets

There are three main datasets that we used to build and validate the grading pipeline: (i) MEASURE 1, (ii) PREVENT, and (iii) SURPASS. Note that PREVENT and SURPASS was kept as a test-only datasets and were never used to train our models.

MEASURE 1: In the phase III MEASURE 1 study (NCT01358175), patients with radiographic axSpA (ankylosing spondylitis) fulfilling the modified New York criteria were randomised (1:1:1) to receive intravenous secukinumab 10 mg/kg (at baseline, week 2 and week 4) followed by subcutaneous secukinumab 150 mg or 75 mg every four weeks, or matched placebo. Placebo-treated patients were re-randomised to subcutaneous secukinumab 150 or 75 mg from week 16. We included 131 patients from the MEASURE 1 study^41^, in which T1 and STIR spinal MRIs were taken at various time points (Baseline, Weeks 16, 52, 104, 156, 208, and some unscheduled scans). The complete dataset included 132 patients and 570 MRI studies (a single scan refers to a combination of T1-weighted and STIR sequences together); a majority of these SIJ scans were graded both by R1 and R2. There are 3 separate read sessions for each reader where the first read session (session 1) covers scans from Baseline to Week 52, the second read session (session 2) covers scans from Baseline to Week 104, while the last read session (session 3) covers all the scans in the dataset. We use 8-fold cross-validation of the whole dataset to train and validate the model; we split on a per-patient basis so that the same patient will only appear on the same split without spilling over to a different set.

PREVENT: In the PREVENT study (NCT02696031), adult patients fulfilling the Assessment of SpondyloArthritis International Society (ASAS) classification criteria for non-radiographic axSpA with elevated CRP and/or MRI inflammation were randomised (1:1:1) to receive subcutaneous secukinumab 150 mg with a loading dose, 150 mg without a loading dose, or placebo at baseline and weeks 1, 2, and 3, followed by monthly dosing starting at week 4; all patients received open-label secukinumab from week 52 onward. We included 555 patients from the PREVENT study^42^ with 2015 MRI studies. The imaging protocol is similar to MEASURE 1 and as such this dataset also possesses multiple time points (Baseline, Weeks 16, 52, 104, and some unscheduled scans). Similar to MEASURE 1, PREVENT also has 2 readers and 3 read sessions. Note that, PREVENT was used as a completely held-out dataset to validate the models built on MEASURE 1.

SURPASS: In the SURPASS study (NCT03259074), patients diagnosed with active AS fulfilling the Modified New York Criteria and with Bath Ankylosing Spondylitis Disease Activity Index (BASDAI) $\ge$ 4 were randomised (1:1:1) to receive secukinumab 150 mg, adalimumab biosimilar 40 mg, or placebo. We included 414 patients from the SURPASS study with 1503 MRI studies^43^. The imaging protocol is similar to MEASURE 1 and PREVENT with multiple time points (Baseline, Weeks 16, 52, and 104). Unlike MEASURE 1 and PREVENT, SURPASS possesses only 1 read session and 3 separate readers. SURPASS is also used as a held-out dataset to validate the model trained on MEASURE 1.

The readers from the three datasets (7 in total) are anonymised and there might readers who appear in all three datasets and as such, as discussed previously, we only use the inter-reader consensus labels, $Consensus_{R1,R2}$, to assess our predictions.

In terms of pre-processing, each slice is resampled to a fixed in-plane resolution of (224 $\times$ 224) pixels. Variations in slice thickness and slice count are not normalised via resampling along the (z)-axis; instead, all available slices within a volume are processed on a slice-wise basis, with region-of-interest (ROI) consistency enforced across slices. Physical measurements are derived using the original DICOM in-plane pixel spacing.

**Table 2:** Number of graded regions in MEASURE-1, PREVENT and SURPASS with negative scoring according to R1, R2, and R3 over three reading sessions (Ankylosis). Note: Reader 1, Reader 2, Reader 3 and Both Readers are unique and different for MEASURE-1, PREVENT, and SURPASS.

		Reader 1				Reader 2				Reader 3	Readers consensus
Dataset	Score	Session 1	Session 2	Session 3	Consensus	Session 1	Session 2	Session 3	Consensus	Session 1	
MEASURE-1	Negative	421	568	539	721	411	559	483	677	–	672
	Positive	205	270	353	363	221	285	407	407	–	351
PREVENT	Negative	0	252	3996	3996	0	6	4028	4028	–	4012
	Positive	0	0	18	18	0	0	0	0	–	0
SURPASS	Negative	1497	–	–	–	1651	–	–	–	1420	1354
	Positive	1509	–	–	–	1341	–	–	–	1582	1240

**Table 3:** Number of graded regions in MEASURE-1, PREVENT and SURPASS with negative scoring according to R1, R2, and R3 over three reading sessions (Sclerosis).

		Reader 1				Reader 2				Reader 3	Readers consensus
Dataset	Score	Session 1	Session 2	Session 3	Consensus	Session 1	Session 2	Session 3	Consensus	Session 1	
MEASURE-1	Negative	549	759	846	1006	547	723	749	903	–	873
	Positive	77	79	46	60	85	121	141	109	–	33
PREVENT	Negative	0	216	3768	3745	0	6	3827	3827	–	3628
	Positive	0	36	246	238	0	0	201	201	–	70
SURPASS	Negative	2490	–	–	–	2729	–	–	–	1824	1773
	Positive	504	–	–	–	263	–	–	–	1180	169

**Table 4:** Number of graded regions in MEASURE-1, PREVENT and SURPASS with negative scoring according to R1, R2, and R3 over three reading sessions (Oedema).

		Reader 1				Reader 2				Reader 3	Readers consensus
Dataset	Score	Session 1	Session 2	Session 3	Consensus	Session 1	Session 2	Session 3	Consensus	Session 1	
MEASURE-1	Negative	2140	3110	3318	4108	2157	3021	3302	3921	–	3817
	Positive	300	234	202	210	379	331	266	267	–	135
PREVENT	Negative	7352	11132	14502	13742	6926	10437	14219	13111	–	12498
	Positive	1328	1484	1578	1087	1730	2171	1893	1538	–	762
SURPASS	Negative	11165	–	–	–	11231	–	–	–	10487	10167
	Positive	843	–	–	–	793	–	–	–	1529	438

**Table 5:** Number of graded regions in MEASURE-1, PREVENT and SURPASS with negative scoring according to R1, R2, and R3 over three reading sessions (Erosion).

		Reader 1				Reader 2				Reader 3	Readers consensus
Dataset	Score	Session 1	Session 2	Session 3	Consensus	Session 1	Session 2	Session 3	Consensus	Session 1	
MEASURE-1	Negative	1872	2549	2727	3161	1521	2181	2651	2711	–	2488
	Positive	624	803	833	806	1007	1195	909	1026	–	577
PREVENT	Negative	0	790	13775	13719	0	24	14128	14128	–	12821
	Positive	0	218	2281	2239	0	0	1984	1984	–	1004
SURPASS	Negative	8470	–	–	–	9023	–	–	–	8299	7088
	Positive	3554	–	–	–	2948	–	–	–	3741	1941

**Table 6:** Number of graded regions in MEASURE-1, PREVENT and SURPASS with negative scoring according to R1, R2, and R3 over three reading sessions (fatty lesion).

		Reader 1				Reader 2				Reader 3	Readers consensus
Dataset	Score	Session 1	Session 2	Session 3	Consensus	Session 1	Session 2	Session 3	Consensus	Session 1	
MEASURE-1	Negative	1011	1611	1513	1734	1145	1727	1810	2039	–	1492
	Positive	1493	1741	2047	2161	1383	1641	1750	1925	–	1778
PREVENT	Negative	0	805	13776	13743	0	24	14324	14324	–	13123
	Positive	0	203	2272	2245	0	0	1780	1780	–	1102
SURPASS	Negative	3640	–	–	–	6938	–	–	–	1065	1009
	Positive	8328	–	–	–	5034	–	–	–	10871	4844

Tables 2,3, 4, 5, and 6 present the distribution of lesion-negative (score = 0) and lesion-positive (scores = 1/2/3) findings for five lesion types, as assessed by readers R1, R2, and R3 across three reading sessions in the MEASURE-1, PREVENT, and SURPASS studies. Results are reported per region: left/right for ankylosis and sclerosis, and left/right quadrants for oedema, erosion, and fatty lesion.

### Results

Table 7 contains the results of the separate lesion-type prediction on all three datasets. The results on MEASURE-1 are specifically on the cross-validation test set and the results on PREVENT and SURPASS are on the full set of data. Overall, the best-performing lesion type prediction is Ankylosis with 0.97 AUC and 0.93 Balanced Accuracy in MEASURE-1 which is verified by the performance of the same task on SURPASS with an AUC of 0.99 and balanced accuracy of 0.97; note that PREVENT, being an early axSpA dataset, does not have ankylosis cases in any of the scans. The performance of the model in predicting sclerosis, erosions, and fatty lesions are similar in MEASURE-1 with AUC values ranging from 0.87 to 0.89 and balanced accuracies ranging from 0.85 to 0.88. This is confirmed again on the held-out datasets, PREVENT and SURPASS, with AUCs ranging from 0.88 to 0.92 in PREVENT and 0.95 to 0.98 in SURPASS. The lowest performing lesion type prediction in MEASURE-1 is oedema classification with 0.85 AUC and 0.79 balanced accuracy. This is also true in SURPASS which shows oedema classification being the lowest performing classification task at 0.92 AUC and 0.86 balanced accuracy compared to the other tasks. This is slightly different in PREVENT where the AUC of 0.91 in oedema classification is higher than sclerosis and erosion classifications.

**Table 7:** Classification results on MEASURE-1, PREVENT, and SURPASS. Performance is reported using area under the ROC curve (AUC), balanced accuracy (BA), sensitivity (Sens.), and specificity (Spec.). Note that there were no cases of ankylosis in PREVENT, as confirmed by annotations from the two expert readers.

Task	MEASURE-1				PREVENT				**SURPASS**			
	AUC	BA	Sens.	Spec.	AUC	BA	Sens.	Spec.	AUC	BA	Sens.	Spec.
Ankylosis	0.97	0.94	0.92	0.95	–	–	–	–	0.99	0.91	0.91	0.91
Sclerosis	0.89	0.71	0.50	0.93	0.88	0.63	0.31	0.96	0.98	0.71	0.56	0.86
Oedema	0.85	0.78	0.60	0.96	0.91	0.79	0.63	0.94	0.92	0.78	0.59	0.96
Erosions	0.87	0.76	0.67	0.86	0.89	0.70	0.48	0.92	0.96	0.80	0.71	0.88
Fatty Lesions	0.89	0.83	0.85	0.82	0.92	0.74	0.55	0.93	0.95	0.71	0.79	0.63

## Discussion

In this study, we developed a fully automatic machine learning—based system to classify the presence or absence of five lesion types in coronal sacroiliac joint (SIJ) MRI scans. Overall, automated scoring of MRI scans in patients with axial spondyloarthritis (axSpA) using the proposed machine learning software was found to be comparable to expert reader–based assessments.

We evaluated the classifier using different labelling strategies. When the model was trained and evaluated using individual reader scores, performance was inferior to that obtained using consensus labels derived across multiple readers and read sessions. The consensus-based approach achieved accuracies ranging from 0.63 to 0.94 across the five lesion classification tasks, which are comparable to inter-reader performance.

In terms of area under the receiver operating characteristic curve (AUC), for oedema prediction, our model achieved an AUC of 0.85 on MEASURE-1, 0.91 on PREVENT, and 0.92 on SURPASS. These results are comparable to the AUC of 0.87 reported by Lin et al. ^44^ for oedema detection in whole-spine MRI, which represents a less challenging classification task. Overall, AUC values across all five lesion types ranged from 0.85 to 0.99, indicating consistently high classification performance. Future work will focus on mitigating the trade-off between label consistency and dataset size. Additional datasets with larger numbers of non-zero oedema scores may enable improved training and validation of the machine learning models. This study has several limitations. To address variability in human labels, we evaluated model performance using reader-consensus subsets (two readers, R1 and R2, for MEASURE-1 and PREVENT; three readers, R1, R2, and R3, for SURPASS) and analysed performance within these subsets. While this strategy improves label reliability, it comes at the cost of discarding a substantial number of samples due to reader disagreement. As shown in Table 7, the highest AUC (0.99) was achieved using the *consensus* subset. However, this improvement was obtained by excluding grades that did not meet consensus criteria. Notably, during training (using MEASURE-1), all reader gradings across all reading sessions were used; only consensus labels were used for evaluation.

## Conclusion

In this paper, we presented an end-to-end pipeline for detecting acute and chronic lesions of the SIJs, starting with contouring/delineating the SIJs, followed by a stage to classify lesions within the extracted SIJs. We independently validated both stages using datasets with a substantial number of SIJ MRIs, achieving high lesion detection performance, which was further corroborated by validation on two completely held-out datasets.

## Data collection authorization

The axSpA datasets used in this study (MEASURE 1, PREVENT, and SURPASS) were collected from completed, anonymised clinical trials. Since these MRI scans were anonymised, they are no longer considered personal data, and therefore, no additional approval from Ethics Committees or Institutional Review Boards (EC/IRBs) is required. The details of the clinical trials, and the necessary approvals, are available as follows: MEASURE 1: NCT01358175, PREVENT: NCT02696031, and SURPASS: NCT03259074.

The scans in the Oxford Sacroiliac Joint (OSIJ) dataset were sourced from retrospective scans in the Oxford University Hospitals as part of Oxford Secondary Care Lumbar Magnetic Resonance Imaging Cohorts (OSCLMRIC), which is a Health Research Authority (HRA) approved study (IRAS Project ID 207858). The University of Oxford is the sponsor of this research, in keeping with the requirements of the UK Policy Framework for Health and Social Care Research 2017. Health Research Authority approval for receipt and analysis of anonymised retrospective patient data was received in 2016 (project reference 207858) to assist in the development of an image analysis methodology to analyse clinical MRI studies in subjects with low back pain syndromes and asymptomatic controls. PID 12139 Protocol Number 12139. Date/version 23/08/2016; v9.0; Minor amendments (to increase scope of recruitment and duration) were requested 18th March 2019) All the subjects in this report had been recruited before this date. IRAS Project ID: 207858 REC Reference: 16/HRA/4532 Short Study.

## Data Availability

The MEASURE 1, PREVENT, and SURPASS datasets were obtained through the Big Data Institute and Novartis research alliance and are owned by Novartis Pharmaceuticals. Access to these datasets can be requested directly from Novartis Pharmaceuticals. The OSIJ scans were extracted and anonymised from local hospital systems under a contract that explicitly prohibits data sharing with third parties for patient privacy reasons; therefore, they cannot be made publicly available.
